# Highly Defective Dark Nano Titanium Dioxide: Preparation via Pulsed Laser Ablation and Application

**DOI:** 10.3390/ma13092054

**Published:** 2020-04-28

**Authors:** Elena D. Fakhrutdinova, Anastasiia V. Shabalina, Marina A. Gerasimova, Anna L. Nemoykina, Olga V. Vodyankina, Valery A. Svetlichnyi

**Affiliations:** 1 Laboratory of Advanced Materials and Technology, Tomsk State University, Tomsk 634050, Russia; fakhrutdinovaed@gmail.com; 2 Laboratory of Biophotonics, Siberian Federal University, Krasnoyarsk 660041, Russia; marina_2506@mail.ru; 3 Laboratory of Biopolymers and Biotechnology, Tomsk State University, Tomsk 634050, Russia; nemoykina@rambler.ru; 4 Laboratory of Catalytic Research, Tomsk State University, Tomsk 634050, Russia; vodyankina_o@mail.ru

**Keywords:** dark titania, pulsed laser ablation in liquid, defects, calcination, nanopowders, photocatalysis, phenol, antibacterial properties

## Abstract

The development of methods to synthesize and study the properties of dark titania is of the utmost interest due to prospects for its use, primarily in photocatalysis when excited by visible light. In this work, the dark titania powder was prepared by pulsed laser ablation (Nd:YAG laser, 1064 nm, 7 ns) in water and dried in air. To study the changes occurring in the material, the thermal treatment was applied. The structure, composition, and properties of the obtained powders were studied using transmission electron microscopy, low-temperature N_2_ adsorption/desorption, X-ray diffraction, thermogravimetry/differential scanning calorimetry, X-ray photoelectron, Raman and UV-vis spectroscopies, and photoluminescence methods. The processes occurring in the initial material upon heating were studied. The electronic structure of the semiconductor materials was investigated, and the nature of the defects providing the visible light absorption was revealed. The photocatalytic and antibacterial activities of the materials obtained were also studied. Dark titania obtained via laser ablation in liquid was found to exhibit catalytic activity in the phenol photodegradation process under visible light (>420 nm) and showed antibacterial activity against *Staphylococcus aureus* and bacteriostatic effect towards *Escherichia coli*.

## 1. Introduction

Titania (TiO_2_) is a multifunctional semiconducting material that has found applications in a wide variety of fields [1,2]. TiO_2_ has three crystal modifications, namely, anatase, rutile, and brookite [3]. Different non-stoichiometric titania phases can be formed, and the Magneli phases Ti_n_O_2n−1_ [4] bring about a great interest. Depending on their structure, the titanium oxides exhibit significantly different properties. Rutile is a rather inert oxide for biological objects and is characterized by rather weak photocatalytic properties towards oxidation of organic compounds. It is also used in cosmetics, food industry, paints and varnishes, etc. [5]. Anatase is more active, features antibacterial properties, and is known as an effective photocatalyst [6,7]. Different titania forms are widely used in biomedicine [8,9].

Normally, titania is a white powder with the band gap energy of 3.0–3.2 eV, and it does not absorb light in the visible range [3] that limits its use in photocatalysis. To increase the effectiveness and widen the spectral range of its photocatalytic activity, doping [10,11,12], core@shell structures’ formation [13], decoration [14], and the formation of complex composite/hybrid oxides [15,16] are considered.

Nowadays, “dark” titania is also of great interest. The reduction can change the color of the initially white oxide. Thus, in Reference [17], polycrystalline titania in a reducing media changed its color from white to yellow, then to brown, and finally became blue-black. The black color is due to the presence of the oxygen vacancies of different types, Ti^3+^ and Ti^4+^ interstitial ions, and Ti–H bonds [18]. Thus, the disorder of the titania lattice plays a significant role in the formation of black TiO_2_. Structural defects lead to the appearance of additional energy levels in the band gap that is a key factor determining the optical and photocatalytic activity of the materials. Dark titanium dioxide was first obtained in 2011 by heating white TiO_2_ at 200 °C and H_2_ pressure of 20.0 bar [19]. To date, there are a number of publications devoted to the preparation and study of black titanium dioxide, including several reviews [20,21,22]. To synthesize black TiO_2_, a number of methods were proposed, including hydrogenation of white titanium dioxide at high and low pressures [21,22], treatment with hydrogen plasma [23], electrochemical and chemical reduction [23,24], and laser irradiation [20,25]. Of great interest is the synthesis of dark oxide from a titanium metal target by pulsed laser ablation in a liquid (LAL).

The high-energy nonequilibrium LAL method is currently used to obtain a wide range of nanoparticles (NPs) [26,27,28,29] and for surface structuring [30,31]. The LAL also allows for the production colloids of dark titanium oxide. To date, many publications have been devoted to the ablation of metallic titanium, e.g., [32,33,34,35,36,37,38,39,40,41,42,43,44,45]. Nevertheless, the studies of titanium LAL and the resulting nanostructures continue to attract great interest. This is due to the wide variability of the method depending on the radiation characteristics, other parameters of the ablation process, and the composition of the solution. By varying the radiation characteristics, the reaction medium, and other experimental parameters, the particles of various sizes, stoichiometry, defect concentrations, and crystal structures can be obtained from a metal target. Even under rather similar experimental conditions, it is possible to obtain titanium dioxide NPs with various properties. Thus, the LAL of Ti in water by the radiation of a Nd:YAG laser (1064 nm) with varying experimental parameters allows for obtaining the crystal structure changes (amorphous, anatase, brookite, rutile) and particles with various defects and non-stoichiometric composition [32,33,34,35,36]. The use of shorter pulses (pico-sec, femto-sec) and wavelengths further diversifies the size, morphology, and structure of the NPs obtained [37,38]. The processes of secondary interaction play an important role. This was confirmed by experiments on the additional laser irradiation of colloids [39,40]. In addition, the properties of the powders obtained by drying the colloids synthesized by LAL were poorly studied.

The present work aims to study the morphology, structure, and optical properties of nanopowders obtained by drying and further thermal treatment of dark titanium oxide colloids synthesized by LAL of metal titanium in water. The photocatalytic and antibacterial properties of the obtained powders are also studied.

## 2. Materials and Methods

### 2.1. Synthesis of the Materials

Initial TiO_2_ nanopowder was prepared according to the following procedure. First, the colloidal solution was obtained by pulsed laser ablation in liquid of titania target (99.9%). The Nd:YAG laser LS2131M-20, LOTIS TII (Minsk, Belarus) was used for the LAL. The ablation was carried out in a 100-mL cylindrical reactor for 3 h. Then, the solution was dried in air at 60 °C. The initial sample was annealed in the temperature range of 200–1000 °C. Detailed equipment and the experiment on the preparation of nanopowders were described in [26,46] and Supplementary Materials.

The obtained samples were denoted as TiO_2__ini, TiO_2__200, TiO_2__400, TiO_2__600, TiO_2__800, and TiO_2__1000.

### 2.2. Characterization of the Materials

The microstructure of the materials was studied using the transmission electron microscope (TEM) HT-7700, Hitachi (Tokyo, Japan). The scanning electron microscopic (SEM) study was carried out on Vega 3H, Tescan (Brno, Czech Republic). Specific surface and pore sizes were determined using low-temperature N_2_ adsorption/desorption according to the BET method on the TriStar II 3020, Micromeritics (Norcross, GA, USA). Prior to the measurements, the samples were degassed under vacuum (10^−2^ Torr) at 200 °C for 2 h (TiO_2__ini sample was degassed at room temperature). Pore size distribution and porosity were calculated from the desorption isotherms by BJH method (Barrett–Joyner–Halenda).

The X-ray diffraction (XRD) method was used to study the phase composition and average regions of coherent scattering of the powder samples. A Shimadzu XRD 6000 (Kyoto, Japan) diffractometer was used to record the diffraction patterns. The phase composition was identified using the PDF-4 database. The phase contents were calculated using the PowderCell 2.4 software complex (Federal Institute for Materials Research and Testing, Berlin, Germany).

Thermal analysis in the region of 25–1000 °C was performed using Netzsch STA 449 F1 Jupiter^®^ (Selb, Germany) with a rate of 10°/min under dry air flow with the rate of 50 mL/min. The samples were placed in alundum (Al_2_O_3_) crucibles.

Raman spectra were recorded using the Raman microscope InVia, Renishaw (Wotton-under-Edge, UK), with laser excitation at a wavelength of 785 nm in the range of 100–1000 cm^−1^ at a power < 1 mW to avoid sample changing during the analysis.

X-ray photoelectron spectroscopy (XPS) data were obtained using X-ray photoelectron spectrometers KRATOS ES 300, Kratos Analytical (Manchester, UK) with a MgKα source.

The UV-Vis absorption spectra (230–800 nm) of the powders were collected using spectrophotometer Cary 100SCAN, Varian (Belrose NSW, Australia) equipped with the accessory DRA-CA-30I, Labsphere (North Sutton, NH, USA). The band gap was calculated from the diffuse reflection spectra using the Tauc method for indirect semiconductors [47]. The photoluminescence (PL) spectra (280–750 nm) of the powder were recorded on a Fluorolog 3–22 spectrofluorometer, Horiba, Jobin Yvon (Edison, NJ, USA).

### 2.3. Photocatalytic and Antibacterial Activity Studies

The photocatalytic activity of the materials was assessed based on phenol degradation by visible light. Metal-halogen lamp Master Colour CDM-TD 70W/942, Philips (Hamburg, Germany), with the cut-off filter YG11 (410 nm) (Figure S1) was used. A portion of 50 mg of the powder under study was dispersed in 50 mL of phenol solution in water (5 × 10^−5^ M). Prior to the photocatalytic experiment, the dark stage was performed for 1 h to reach the sorption equilibrium. After this, the irradiation was carried out for 1 h at a constant stirring by a magnet stirrer. The luminosity on the surface of the solution was 66 klx (direct sunlight is characterized with 30–120 klx).

Photodegradation and sorption values were calculated based on the phenol concentration decrease. The phenol concentration was determined using fluorimetry from the change in the phenol luminescence intensity at a wavelength of 300 nm using a CM2203 spectrofluorometer, SOLAR (Minsk, Belarus). The phenol concentration calibration was preliminarily performed. Changes in the absorption spectra of the solutions were monitored photometrically with a Cary 100SCAN spectrophotometer. The particles were removed from the solution via ultracentrifugation by centrifuge Allegra 64R, Beckman Coulter (Brea, CA, USA) for 30 min at 20,000 rpm.

The antibacterial activity of the dark titania was tested over two bacteria, namely, gram-positive *Staphylococcus aureus* (*S. aureus*, test strain ATCC 25923) and gram-negative *Escherichia coli* (*E. coli*, test strain B-6954, Russian Collection of Microorganisms) according to the standard ISO 20743:2013 [48]. The experimental conditions and the procedure of the antibacterial activity measurement are described in detail in our previous work [46]. The NPs from the initial colloid of dark titania were precipitated onto a cotton fabric 120 g/m^2^ TexLine. The values of antibacterial activity A were determined as:

(1) A=F−G

where F and G are the growth rates of the control sample and the one loaded with TiO_2_ NPs, respectively.

## 3. Results and Discussion

### 3.1. Structural Studies

#### 3.1.1. Microscopic Data

**Figure 1:**
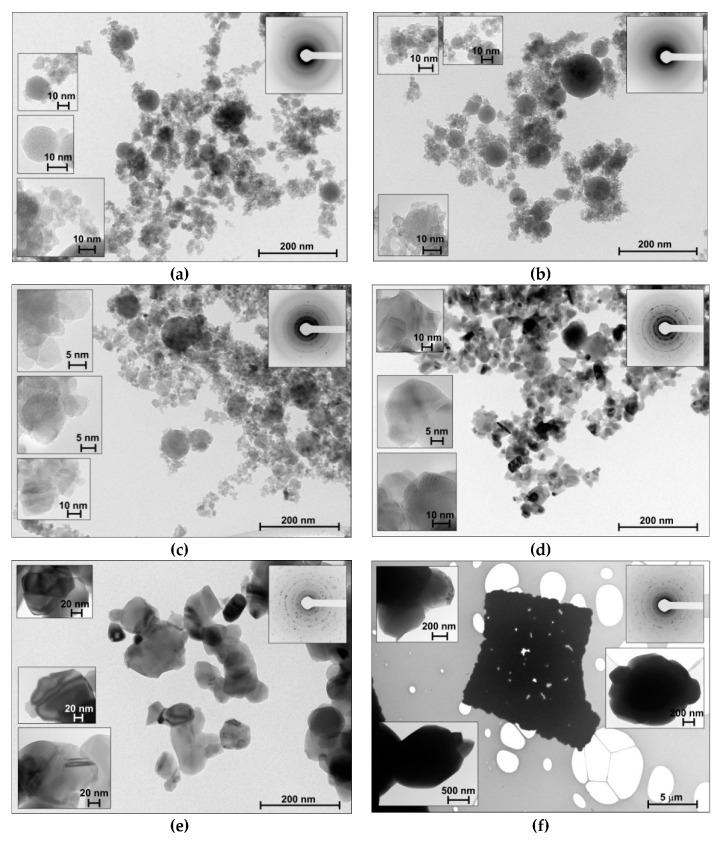
TEM data for the materials: (**a**) TiO_2__ini, (**b**) TiO_2__200, (**c**) TiO_2__400, (**d**) TiO_2__600, (**e**) TiO_2__800, (**f**) TiO_2__1000. Right top corner insets: Selected area electron diffraction (SAED) data.

The initial sample was obtained via LAL followed by drying in air. Then, it was annealed at different temperatures in the region of 200–1000 °C. The initial powder was dark. After 200 and 400 °C, the color changes to light grey, at 600 °C it becomes white, and after heating up to 800 or 1000 °C, the sample is light yellow. To reveal the structural changes of the material during the thermal treatment, the microscopic studies were applied. Figure 1 shows the TEM images of the materials obtained via LAL and subsequent thermal treatment.

The as-prepared sample (Figure 1a) comprises small spherical particles with sizes of ~5–10 nm, and larger particles of 80 nm. Such a bimodal size distribution is quite characteristic for the NPs obtained via LAL [39,41]. This is due to the specific features of the particle formation mechanism under non-equilibrium conditions when the laser beam interacts with the solid target in the liquid media [28].

Thermal treatment changes the size and shape of the particles. The size increases due to sintering. The spheres coalesce to form agglomerates of the irregularly shaped particles. Starting with the TiO_2__400 sample, the particles become facetted. According to the SAED data, the crystallinity increases; TiO_2__ini (Figure 1a) and TiO_2__200 (Figure 1b) samples exhibit amorphous halos, but TiO_2__400 (Figure 1c) and other samples demonstrate the SAED characteristics of polycrystallites. After 800 °C (Figure 1e,f), the particles lose individual boundaries to yield quite dense large crystalline objects. The TiO_2__1000 sample contains individual large flat agglomerates up to 10 μm in size consisting of fused particles (Figure 1f).

#### 3.1.2. Textural Characteristics

Figure S2 and Table 1 show the results on the specific surface area and porosity. It is noteworthy that the isotherms for the initial sample and the materials after the thermal treatment up to 600 °C are of the same type. These samples also demonstrate similar porous structures. Since the pores in the samples are formed by the voids between the particles, the total porosity directly depends on their size and shape. Hysteresis loops at lower relative pressures (from 0.50 to 0.99) indicate the mesoporous structure of the samples TiO_2__ini, TiO_2__200, TiO_2__400, and TiO_2__600. The pore size distribution for these samples is relatively narrow. The initial titania sample exhibits a pronounced single peak that is located at 7.5 nm and shifts as the temperature of the thermal treatment increases. The sample TiO_2__800 shows the matching of the adsorption and desorption isotherms indicating the absence of pores in the sample. Such isotherms cannot be registered for the TiO_2__1000 sample.

Specific surface area and pore volume values were calculated from the desorption isotherms obtained (Table 1). The initial sample demonstrates the largest S_BET_ of 227 m^2^/g. The specific surface smoothly decreases with the increase in the heating temperature up to 600 °C. A further temperature increase leads to a sharp decline in the specific surface area and porosity. These results are consistent with the TEM data.

Thus, the materials obtained comprise the spherical amorphous particles growing under thermal treatment that increases the crystallinity and decreases the specific surface area and pore volume of the samples.

### 3.2. Composition Study

#### 3.2.1. XRD Data

**Figure 2:**
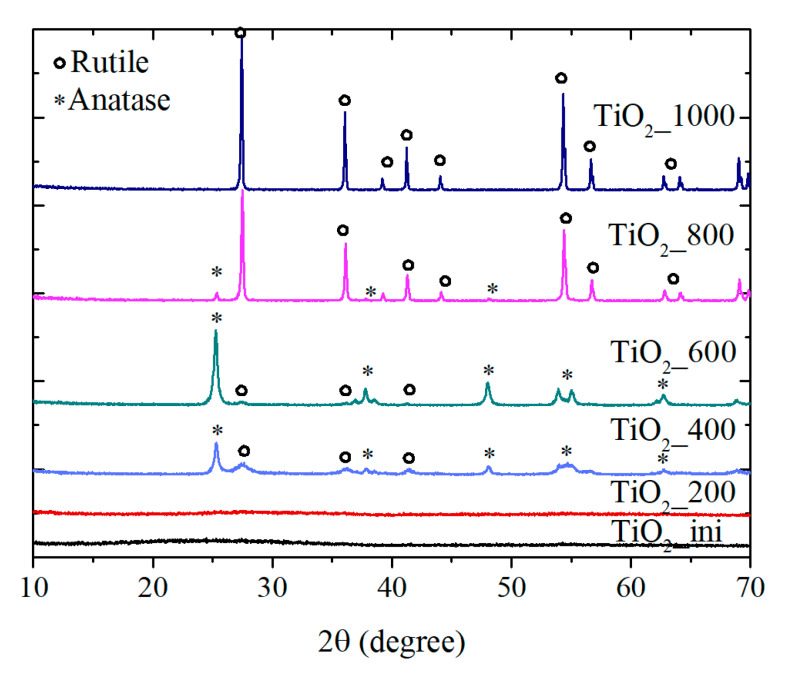
XRD patterns of the samples.

Figure 2 shows the XRD patterns of the samples. The initial material is amorphous that is consistent with the SAED data. After treatment at 200 °C, the first inclusions of the anatase and rutile crystal phases can be observed, even though the sample is still amorphous. After heating at 400 and 600 °C, the amorphous part of the samples disappeared completely, and anatase became the major phase (Table 1). A further temperature increase leads to the anatase-to-rutile transition.

The anatase/rutile phase transition of the bulk material is known to start at 450 °C, and, at 750 °C, the pure rutile phase is formed [49]. In the case under study, the anatase/rutile transition takes place at higher temperatures. Thus, the materials obtained are characterized by the enhanced stability to the thermally induced phase transition. This may be explained by the defects in the surface and sub-surface layers that were formed because of the fast cooling of the substance during the LAL process. These defects seem to act as a hindrance for the fast sintering of the particles that significantly appears only at 800 °C (see the TEM data, Section 3.1.1., and Table 1).

Thus, the NPs obtained via LAL are amorphous. They crystallize during the thermal treatment, and anatase-to-rutile transition occurs at higher temperatures pointing at higher phase stability of the material. To shed some light on the state and the crystallization process of the initial material, the thermogravimetry/differential scanning calorimetry (TG/DSC) and Raman studies were performed.

#### 3.2.2. TG/DSC Study

**Figure 3:**
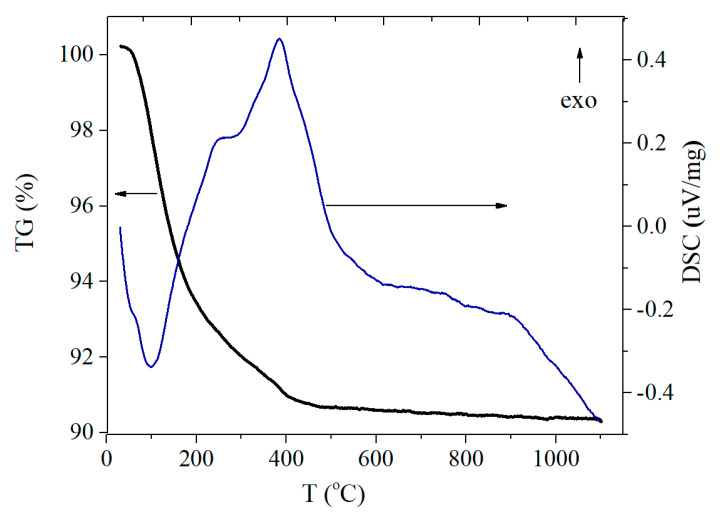
Thermogravimetry/differential scanning calorimetry (TG/DSC) curves of TiO_2__ini.

Figure 3 shows the results of the TG/DSC analysis of the initial LAL-obtained sample.

Two different processes are seen at the DSC curve. The endo-thermal process in the temperature range of 40–200 °C and the mass loss on the TG curve are associated with the removal of physically adsorbed and chemically bound water. The further slight decrease in mass up to 400 °C is probably due to the removal of carbonates from the surface of the particles. The exothermic effect on the DSC curve in the temperature range of 250–450 °C correlates with the crystallization of the initially amorphous TiO_2_. Above 600 °C, the structural transformation of anatase to rutile begins that refers to the endo-thermal process.

The process of amorphous TiO_2_ transition into the crystalline one normally occurs at 350–400 °C [50,51]. We believe that such an early crystallization is connected with the small particle size and the specificity of the amorphous material obtained under LAL. For instance, the surface and sub-surface defects mentioned in the previous section might be responsible for it. To receive more information on the structure of the as-obtained TiO_2__ini and TiO_2__200, Raman spectroscopy was applied.

#### 3.2.3. Raman Spectroscopy Studies

**Figure 4:**
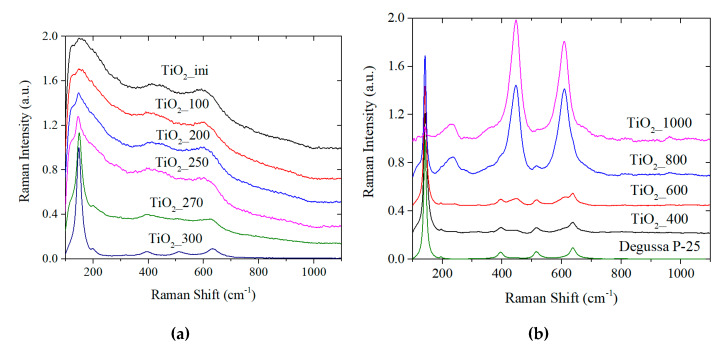
Raman spectra of the samples annealed under different temperatures: ini–300 °C (**a**) and 400–1000 °C (**b**).

Figure 4 shows the Raman spectra for all samples considered. To study the amorphous state of the initial samples, the spectra for the samples after heating at 100, 250, 270, and 300 °C were additionally recorded. For the powders annealed at temperatures up to 250 °C, three wide weak bands can be observed. The short-wavelength band at ~150 cm^−1^ belongs to the base mode Eg for anatase. The other bands with the maxima at ~420 and 600 cm^−1^ are characteristic for Eg and A1g of rutile, respectively [52,53]. A thermal treatment temperature increase of up to ~270 °C leads to the formation of anatase structure (Figure 4a). After heating at 300 °C, the Raman spectrum contains only bands at 144 and 196 cm^−1^ (Eg mode), 398 (B1g), 513 (A1g) and 637 (Eg) characteristic for anatase [52].

Weak bands of the rutile phase are present in the spectrum for the initial sample. The characteristic anatase short-wavelength band is more intensive but some inhomogeneity (appearing in different intensity and wideness) is observed. The initial sample may consist of a mixture of fine crystallites of anatase and rutile that have only short-range order. Thermal treatment leads to the anatase crystal lattice completion, which is consistent with the DSC and XRD data.

A further temperature increase results in narrowing of the anatase bands (Figure 4b) that indicates the enlargement of the particles [54]. Then, starting from 600 °C, in addition to the bands belonging to anatase, the rutile vibrational bands (236 (B1g), 446 (Eg) and 610 (A1g) cm^−1^ [55,56]) appear in the spectra. After the calcination at 800 °C, rutile modes dominate in the spectra. Treatment at 1000 °C results in almost complete disappearance of the anatase modes from the spectra. This data is consistent with the XRD results.

Thus, the NPs of TiO_2_ obtained via LAL presumably consist of fine anatase and rutile crystallites that form pure anatase and then rutile phases under thermal treatment. The material shows the shifting of the characteristic phase transition temperatures. We believe this is due to the presence of defects in the surface and sub-surface layers. To find out more information on these defects, the XPS and optical studies were performed.

### 3.3. Surface States and Functional Properties Studies

#### 3.3.1. X-Ray Photoelectron Spectroscopy Data

According to the XRD, Raman, and DSC data, the initial sample undergoes structural changes upon annealing to 400 °C associated with the construction of the anatase crystal structure from the X-ray amorphous particles. It is of interest to establish the state of titanium and surface defects associated with the oxygen vacancies during the annealing in this temperature range. Table 2 represents the O 1s/Ti 2p ratio for the materials. The higher the temperature, the lower the ratio. This is connected with the presence of the OH groups of the adsorbed water removed from the surface that is consistent with the DSC data (Section 3.2.2.).

**Figure 5:**
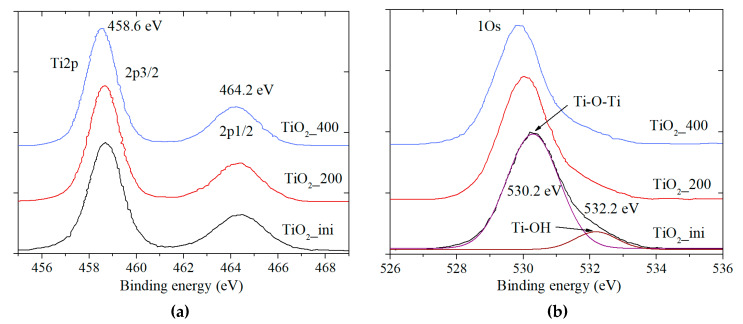
XPS data for the (**a**) Ti 2p and (**b**) O 1s spectra.

The Ti 2p spectra (Figure 5a) contain only a doublet with the binding energy of 458.5 eV (Ti 2p3/2) belonging to the Ti^4+^ state. Other possible titanium states (Ti^3+^, Ti^2+^) are not detected. Thus, it can be concluded that titanium is present in the materials as titania. Figure 5b shows the photoemission spectra of O 1s level. Deconvolution results in two peaks with the binding energies of 529.7–530.2 and 531.9 eV. The first of them belongs to the TiO_2_ lattice oxygen, and the second one is for the oxygen adsorbed on the titania surface.

In addition to titanium and oxygen, carbon exists on the surface, and its concentration decreases with the increasing of the annealing temperature (Table 2). This is consistent with the mass loss in TG/DSC data in the range of 200–400 °C. The presence of carbon can be explained by the CO_2_ sorption from air onto highly active pure particles during drying with the formation of carbonates and bicarbonates on the surface. Similar data on the carbon presence were obtained for other oxides synthesized by LAL [57].

Thus, the XPS method did not detect the specific titanium states (Ti^3+^, Ti^2+^) at the surface of the materials obtained. The defects assumed earlier may demonstrate themselves in the optical properties of the materials.

#### 3.3.2. UV-Visible Absorption Spectra

**Figure 6:**
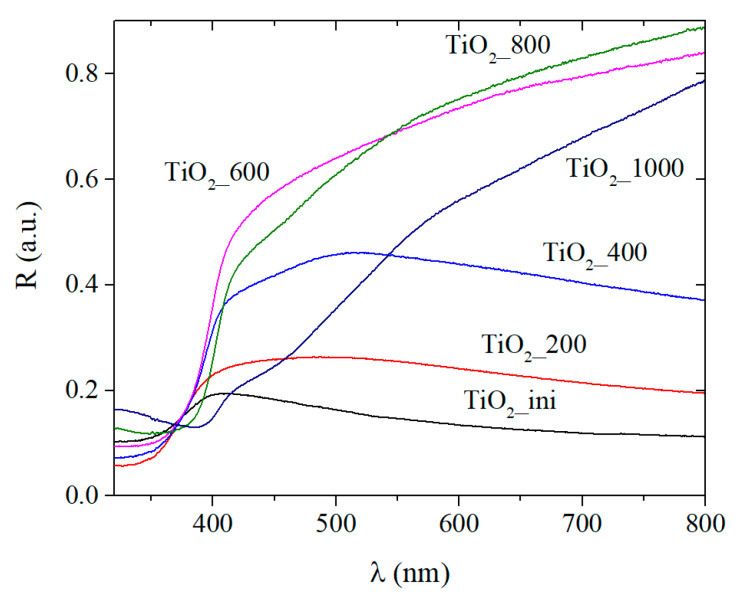
UV-Vis spectra of titania powder.

Figure 6 represents the UV-Vis absorption spectra of the samples. Besides the UV absorption, all samples have additional absorption in the visible region. The initial sample exhibits the most intensive additional absorption in the long-wavelength region. This is probably due to the defective states of different nature. The amount of these defects decreases during the annealing resulting in the intensity decrease for the additional absorption (curves for the TiO_2__400 and TiO_2__600 samples). However, with the subsequent heating temperature increasing to up to 800 and 1000 °C, this additional absorption reappears. The TiO_2__800 and TiO_2__1000 samples are yellow. Normally, titania powder of any modification is white, and it turns yellow upon heating, turning back to white after cooling. Thus, in the case of the samples annealed at 800 and 1000 °C, the Magneli phase [4] is probably formed due to the crystal lattice restructuring accompanied by the number of oxygen atoms change. This point requires further deeper investigation.

Table 3 presents the calculated bandgap values from the DRS spectra (Figure S3) for the samples under study. It is known that bulk titania exhibits the E_g_ of 3.2 eV for anatase and 3.0 eV for rutile. The samples obtained via LAL and annealing demonstrate the decreased E_g_ value. This confirms the presence of energy levels of different defective states in the TiO_2_ bandgap. These levels lead to the blurring of the boundary of valence and conduction bands and “narrow” the bandgap.

Thus, the materials obtained exhibit the decreased bandgap energy and defective structure that is a potential advantage for photocatalysis and other applications. To reveal the nature of the defects more carefully, the photoluminescent properties of the materials were studied.

#### 3.3.3. Photoluminescence Data

**Figure 7:**
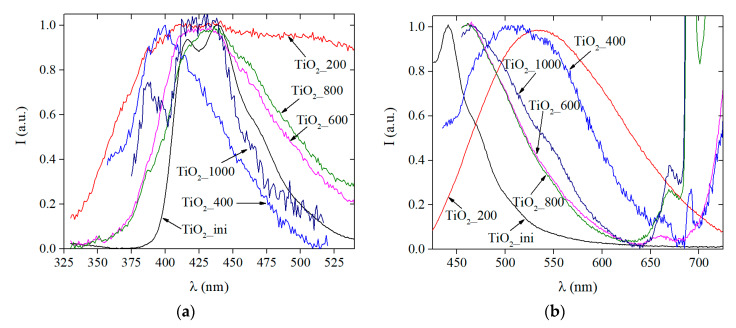
Photoluminescence spectra of titania powders: λ_ex_ = 300 nm (**a**) and λ_ex_ = 405 nm (**b**).

Figure 7 shows the PL spectra of all samples upon excitation either into the exciton absorption band of titanium dioxide, λ_ex_ = 300 nm (Figure 7a), or into the absorption region of defective states (Figure 6 and Figure S3), λ_ex_ = 405 nm (Figure 7b). The shapes of the visible spectra upon excitation at 300 and 405 nm are similar, which indicates a significant contribution to the photoluminescence of structural defects of different nature. To analyze the defects, the published data on the luminescent properties of defects of various types were summarized in Table 4.

The TiO_2__ini sample has a relatively narrow PL spectrum in the region of 300–500 nm. The most intense band with a maximum at 440 nm can be attributed to the excitons localized on TiO_6_ octahedra (the so-called self-trapped excitons, STE) [58,59,60,61,62,63,64]. The STE states appear when an electron traps a hole in a lattice site. It is believed that the STE states are localized around the oxygen vacancies; therefore, they are in the band gap, but close to the valence band of titanium dioxide [61]. The shoulder at 470 nm can be attributed to the oxygen vacancies of various types, the so-called F and F^2+^ centers [58,59,60,64,65,66].

For the TiO_2__200 sample, the PL spectrum becomes much wider (400–700 nm). In the spectrum, the STE states can be distinguished in the region of 400–450 nm. To confirm the presence of the STE states in this sample, we performed a detailed PL experiment with a sequential excitation by different wavelengths (Figure S4). The intense bands in the 450–620 nm region relate to oxygen vacancies of various types (F, F^+^, and F^2+^ centers): F and F^2+^ centers appear in the region of 460–470 nm, F^+^ centers are at 520–540 nm [58,59,60,64,65,66]. Luminescence in the range of 480–495 nm can be attributed to charge transfer from Ti^3+^ or to the oxygen anion in the octahedron [60,61,64] or to the sub-surface oxygen vacancy [67]. The luminescence with the wavelength longer than 600 nm can be associated with the electronic transitions involving Ti^3+^ ions located in different positions of the crystal lattice (sites, internodes) and with various types of oxygen vacancies [65]. It might also belong to the intervalent charge transfer between Ti^3+^/Ti^4+^, where the electron is distributed between the cations on the adjacent interstitial and octahedral positions [65,68].

Upon excitation in the region of the defects (Figure 7b), the TiO_2__400 sample also receives a wide luminescence profile. However, the bands related to the STE states (450 nm) are more pronounced in the spectrum, and a luminescence band longer than 650 nm begins to appear.

The samples annealed at 600, 800, and 1000 °C are also characterized by the PL in the region of 465–470 nm and an intense luminescence longer than 650 nm. The intensity of the long-wavelength PL increases with the increasing of the annealing temperature. A decrease in the intensity of the bands in the region of 550–620 nm probably indicates a significant decrease in the amount of the F^+^ centers. The appearance of the intense long-wavelength luminescence correlates with the long-wavelength absorption in the 400–600 nm region (Figure 6 and Figure S3).

Thus, the nature of the defects present in titania obtained via the LAL was revealed. The majority of the defects are oxygen vacancies. It was also found that under irradiation, these vacancies participate in the formation of the specific STE states. Both these types of defects affect the bandgap value and may play an important role in the material application in photocatalysis, disinfection, etc.

### 3.4. Potential Practical Applications

#### 3.4.1. Photocatalytic Activity Data

**Figure 8:**
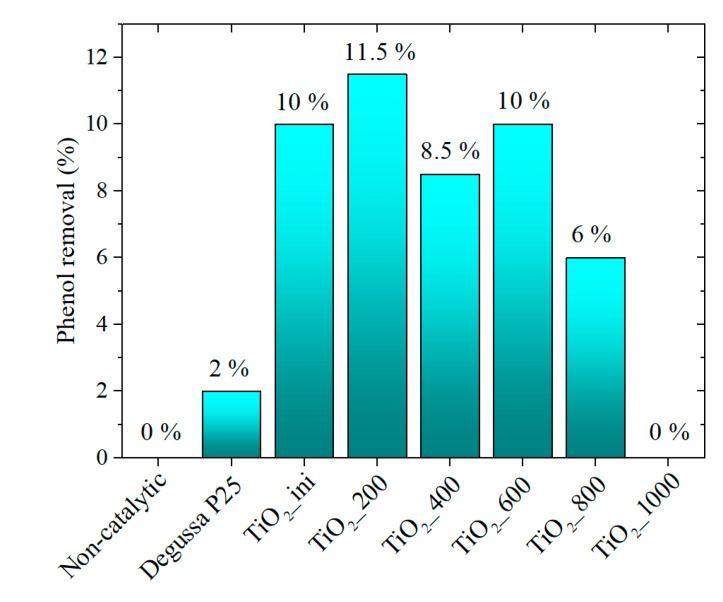
Photodegradation of phenol in water under visible light.

One of the possible applications of the dark titania obtained via LAL is a photocatalytic decomposition of the organic compounds in water. The challenge is to decompose stable organic molecules like phenol under visible light (e.g., sunlight simulation). Thus, to estimate the photocatalytic potential of the materials synthesized in this work, they were tested in phenol photodegradation in water under visible light. The photoactivity of the materials was compared with the commercial titania (Degussa P25). For all samples, the phenol sorption onto the titania surface was less than 1%. Figure 8 shows the results of phenol decomposition during photocatalysis for 1 h of exposure. The phenol decomposition was estimated from the decrease in the fluorescence intensity (fl = 290 nm) upon excitation of the maximum of absorption band (fl = 275 nm). Figure S5a represents the example of changing in the fluorescence spectra. The estimation of photodecay from the absorption spectra is difficult since upon irradiation the intermediate products are formed that absorb in the same region as phenol (Figure S5b). The absorption bands are typical for transitional phenolic intermediates, i.e., p-benzoquinone (246 nm), catechol (270 nm), and hydroquinone (289 nm) [69]. After prolonged exposure, these products are further decomposed into CO_2_ and H_2_O [69]. A more detailed study of the phenol photodegradation products will be performed in our further work.

Without any catalyst, phenol does not decompose even under irradiation. For the case of the P25 commercial powder this process takes place but is rather low-intensive. The samples obtained by LAL demonstrate good catalytic activity. The TiO_2__200 sample seems to be the most active one. This is due to the high specific surface area, the absorption in the visible region (Figure 6), and the corresponding types of defects (Figure 7). Despite an increase in the rutile content and a decrease in the specific surface area, the samples remain active up to an annealing temperature of 800 °C. This is due to both their absorption properties and the defective structure.

Thus, the materials obtained via LAL and via LAL followed by heating at 200 °C exhibited photocatalytic activity in the process of phenol degradation under visible light radiation. These materials are promising photocatalysts to be investigated further.

#### 3.4.2. Antimicrobal Activity Studies

Titania is widely used as an antimicrobial agent since it is characterized by the resistance to high temperatures, low solubility, high specific surface area, and strong oxidizing properties. The antibacterial activity of the materials obtained in this work was tested. TiO_2__ini penetrates well into the cotton fabric when impregnated with a suspension (Figure S6a,b). Table 5 represents the results obtained for the test of the cotton fabric covered with the TiO_2_ particles of various titania content against gram-positive staphylococcus (*S. aureus*) and gram-negative coli bacillus (*E. coli*).

According to the results obtained, staphylococcus has demonstrated higher sensitivity to the presence of TiO_2_ than *E. coli* bacillus, which is consistent with the literature reports [70]. The higher the titania concentration, the higher the antibacterial effect towards *S. aureus*. At the same time, the largest concentration of TiO_2_ particles exhibited only the bacteriostatic effect on *E. coli*. This variation of titania effect may be connected either with different cell membranes structure of the bacteria under study [71,72] or with the contribution of the electrostatic forces between the positively charged cell walls of S. aureus and negatively charged centers on TiO_2_ surface (oxygen vacancies). However, the last point requires additional detailed investigation.

Thus, titania samples obtained via LAL exhibit antibacterial and bacteriostatic effects and may be potentially used as a component of the special bandages, antibacterial coatings, etc. It is noteworthy that titania has an additional potential to increase antibacterial activity [73] as well as anti-cancer activity [74] by means of photoactivation.

## 4. Conclusions

Dark titania powder was obtained via the pulsed laser ablation of metallic titanium in water and subsequent drying. The initial material consisting of fine anatase and rutile crystallites (5–10 nm) is mesoporous with a high specific surface area (227 m^2^/g). The TiO_2_ was found stable under thermal treatment demonstrating the shifts of the phase transitions towards higher temperatures. The material exhibited the intensive visible light absorption due to the additional defect levels in the band gap. For the first time for such materials, the nature of the defects was studied in detail using different methods. The presence of the oxygen vacancies of three types (F, F^+^, and F^2+^ centers) and the STE states was revealed by photoluminescence spectroscopy. It was found for the first time that the irradiation of the material with light with wavelength energy exceeding the anatase bandgap energy value activated the STE states. The lifetime of these states was quite long and they appeared in the PL spectra, resulting in a blue shift.

The changes in the dark titania powders during the thermal treatment were studied in detail. The materials obtained consisted of anatase or rutile or their mixture depending on the temperature applied. The structural characteristics of the particles were changed as well varying the heating conditions. It was shown that the annealing temperature increase led to a decrease in the number of defects that affected the color and absorption ability of the samples. The dark titania obtained via LAL exhibited catalytic activity in the phenol photodegradation process under visible light and showed antibacterial activity against *S. aureus* and bacteriostatic effects towards *E. coli*.

## Figures and Tables

**Table 1:** Nanoparticles’ (NPs’) size, BET, and X-ray diffraction (XRD) data for the samples.

Sample	Average Size (nm) from TEM Data	S_BET_ (m^2^/g)	V_pore_ (cm^3^/g)	Phase Composition (%)		Δd/d × 10^−3^
				Anatase	Rutile	
TiO_2__ini	10	227 ± 23	0.41 ± 0.02	–	–	–
TiO_2__200	14	124 ± 12	0.37 ± 0.02	–	–	–
TiO_2__400	16	86 ± 9	0.37 ± 0.02	67	33	1.5
TiO_2__600	34	50 ± 5	0.28 ± 0.02	62	38	1.1
TiO_2__800	77	7 ± 1	0.06 ± 0.01	3	97	0.2
TiO_2__1000	400	>1	–	–	100	–

**Table 2:** Data from the XPS spectra.

Sample	Carbon Content (%)	O 1s/Ti 2p Ratio
TiO_2__ini	17.4	2.67
TiO_2__200	17.2	2.58
TiO_2__400	14.4	2.44

**Table 3:** Band gap values for the samples determined at (F(R)hυ)^1/2^.

Sample	E_g_, eV (Tauc)
TiO_2_-ini	3.12
TiO_2_-200	3.15
TiO_2_-400	3.00
TiO_2_-600	3.05
TiO_2_-800	2.96
TiO_2_-1000	2.80

**Table 4:** Interpretation of the PL spectra bands.

PL Band	Spectral Range (nm)
STE	400–450 (3.09–2.75 eV) Y. Lei 2001 [58]; 537 (2.3 eV) 431 (2.88 eV) B. Choudhury 2014 [59]; 429 (2.89 eV) B. Choudhury 2013 [60]; 430 (2.88 eV) C. P. Saini 2017 [61]; 400–450 (3.09–2.75 eV) M. V. Dozzi 2017 [62]; 416 (2.97 eV) W-Yu. Wu 2009 [63]; 425 (2.9 eV) S. Paul 2014 [64].
F or F^2+^-center	420–450 (2.95–2.55 eV) V.N. Kuznetsov 2009 [65]; 468 (2.65 eV) B. Choudhury 2014 [59]; 485 (2.71 eV) B. Choudhury 2013 [60]; 465 (2.67 eV) Y. Lei 2001 [58]; 440–456 (2.81–2.71); 471 (2.63 eV) H. Zhang 2014 [66]; 457 (2.71 eV) S. Paul 2014 [64].
F^+^-center	540–619 (2.30–2.00 eV) V.N. Kuznetsov 2009 [64]; 525 (2.36 eV); 500 (2.48 eV) B. Choudhury 2014 [59]; 525 (2.36 eV) Y. Lei 2001 [58]; 531 (2.33 eV) H. Zhang 2014 [66]; 537 (2.31 eV) S. Paul 2014 [64].
Ti^3+^	485 (2.55 eV) C. P. Saini 2017 [61]; 490 (2.53 eV) S. Paul 2014 [64]; 480 (2.58 eV) J. Liu 2008 [67]; 491 (2.52 eV) B. Choudhury 2013 [60].

**Table 5:** Antibacterial activity of dark titania towards *S. aureus* and *E. coli*.

C on Tissue (mg/cm^2^)	The Level of Growth		Antibacterial Activity A
	Control F	Sample G	
*S.aureus* (+)			
0.1	+2.8	+2.8	0
0.25	+2.8	+2.6	+0.2
1	+2.9	–2.9	+5.8
*E.coli* (−)			
1	+2.1	+1.0	+1.1

## Supplementary Materials

The following are available online at https://www.mdpi.com/1996-1944/13/9/2054/s1, Figure S1: Spectrum of UV-Vis lamp Master Colour CDM-TD 70W/942 (Philips, Amsterdam, The Netherlands) with a cut filter (YG11, Tokyo, Japan), Figure S2: Pore size distribution and nitrogen adsorption-desorption isotherms, Figure S3: UV-vis spectra of powder for their band gap calculation, Figure S4: Photoluminescence spectra of the TiO_2__200 sample with the excitation by different wavelengths, while the wavelength was changed in different directions (decreasing or increasing), Figure S5: Fluorescence (a) and absorption (b) spectra of phenol before end after photocatalysis with TiO_2__200, Figure S6: SEM images of cotton (a) and cotton loaded with 0.25 mg/cm^2^ TiO_2_ (b) samples.
