# Supplementary material for: Highly Defective Dark Nano Titanium Dioxide: Preparation via Pulsed Laser Ablation and Application
Source: Materials (Basel). 2020 Apr 28;13(9):2054. doi: 10.3390/ma13092054 (PMC7254401; doi:10.3390/ma13092054)
Supplement: Supplementary file 1 [file materials-13-02054-s001.pdf]

materials

Supplementary Materials

Highly Defective Dark Nano Titanium Dioxide: Preparation via Pulsed Laser Ablation and Application

Elena D. Fakhrutdinova [1], Anastasiia V. Shabalina [1,*], Marina A. Gerasimova [2], Anna L. Nemoykina [3], Olga V. Vodyankina [4] and Valery A. Svetlichnyi [1,*]

[1] Laboratory of Advanced Materials and Technology, Tomsk State University, Tomsk 634050, Russia; fakhrutdinovaed@gmail.com
[2] Laboratory of Biophotonics, Siberian Federal University, Krasnoyarsk 660041, Russia; marina_2506@mail.ru
[3] Laboratory of Biopolymers and Biotechnology, Tomsk State University, Tomsk 634050, Russia; nemoykina@rambler.ru
[4] Laboratory of Catalytic Research, Tomsk State University, Tomsk 634050, Russia; vodyankina_o@mail.ru
* Correspondence: shabalinaav@gmail.com (A.V.S.); v_svetlichnyi@bk.ru (V.A.S.); Tel.: +7-3822-531-591 (A.V.S.)

TiO_2 Powders Synthesis Description

The initial colloidal solution was obtained by the ablation of a plate (10 × 25 × 1 mm) of titanium metal (99.5%) in distilled water. The LAL was performed using the Nd:YAG laser LS2131M-20 (LOTIS TII). The laser had the following characteristics: λ_{las} = 1064 nm, τ_{puls} = 7 ns, E_{puls} = 180 mJ, and f_{rep} = 20 Hz. Ablation in the cylindrical 100-mL glass reactor continued for 3 h. No stirring of the solution during ablation was introduced. Laser radiation was focused through the sidewall of the reactor using a short-focus lens F = 50 mm. The distance between the target and the inner side of the reactor wall was 4 mm. The average diameter of the laser beam on the target surface was 2.5 mm. The maximal radiation power density was ~ 520 MW/cm^2 without taking into account the nonlinear absorption and scattering. We started irradiation at E_{puls} = 100 mJ (~ 300 MW/cm^2) and gradually increased the pulse energy to up to 180 mJ during the first 40 min of ablation (by 10 mJ every 5 min). In this way, we partially compensated for the loss of the radiation intensity due to the nonlinear absorption and scattering.

In order to ensure the uniform irradiation and prevent the formation of craters on the target surface, it was automatically moved in the XY plane orthogonal to the laser beam by two linear stepper motor translation stages (Standa, model 8MT173-50). The scanning was carried out according to the “snake” scheme with a rate of 0.5 mm/sec. The concentration of particles in the dispersion was controlled on the basis of the target mass loss.

To obtain a powder, the colloid was dried in the air in an open glass vessel at 60 °C. Then, the process of the colloid synthesis by LAL and drying was repeated the desired number of times. Then, 3 g of nanocrystalline powder was obtained.

Several portions of the sample were annealed in the muffle furnace at temperatures in the range of 200–1000 °C. The heating rate was 10 °C/min, and the samples were exposed at a given temperature during 4 h.

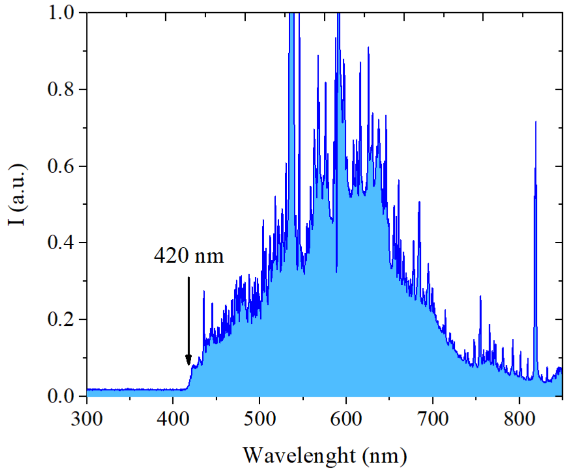

Figure S1. Spectrum of UV-Vis lamp Master Colour CDM-TD 70W/942 (Philips, Amsterdam, The Netherlands) with a cut filter (YG11, Tokyo, Japan).

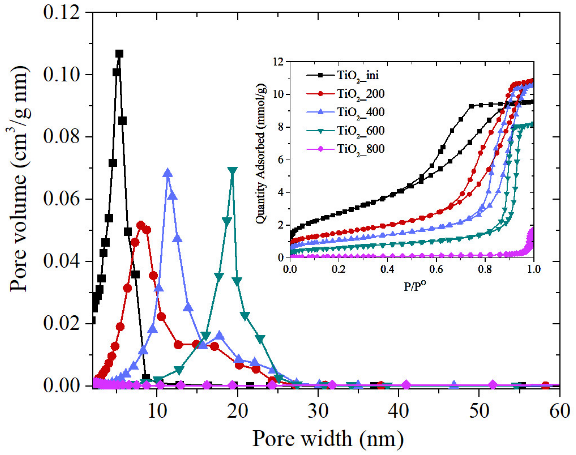

Figure S2. Pore size distribution and nitrogen adsorption-desorption isotherms.

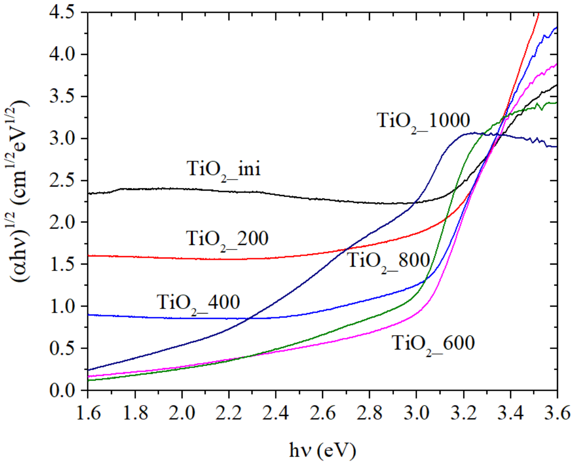

Figure S3. UV-vis spectra of powder for their band gap calculation.

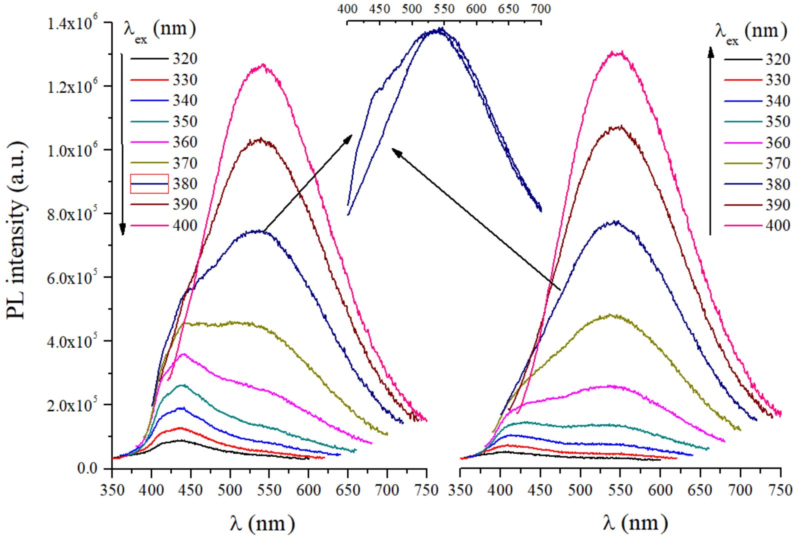

Figure S4. Photoluminescence spectra of the TiO_2_200 sample with the excitation by different wavelengths, while the wavelength was changed in different directions (decreasing or increasing).

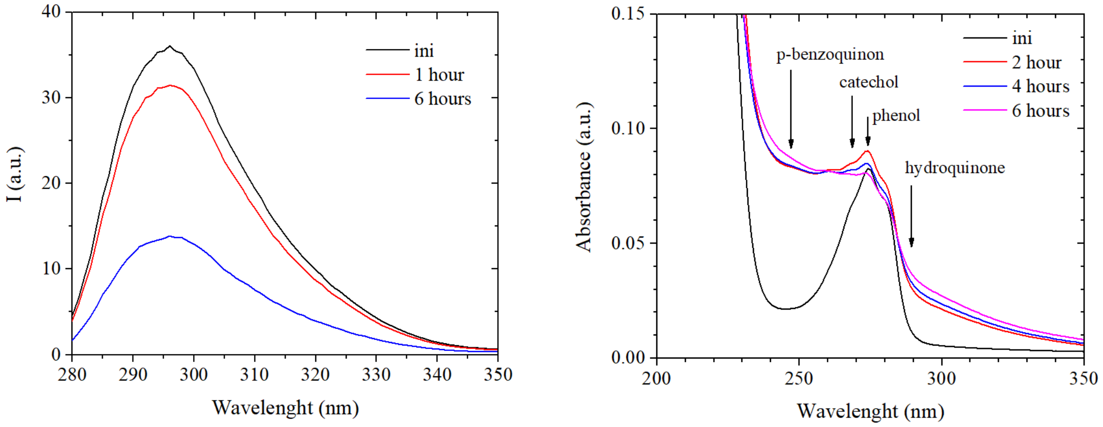

(a) (b)

Figure S5. Fluorescence (**a**) and absorption (**b**) spectra of phenol before end after photocatalysis with TiO_2_200.

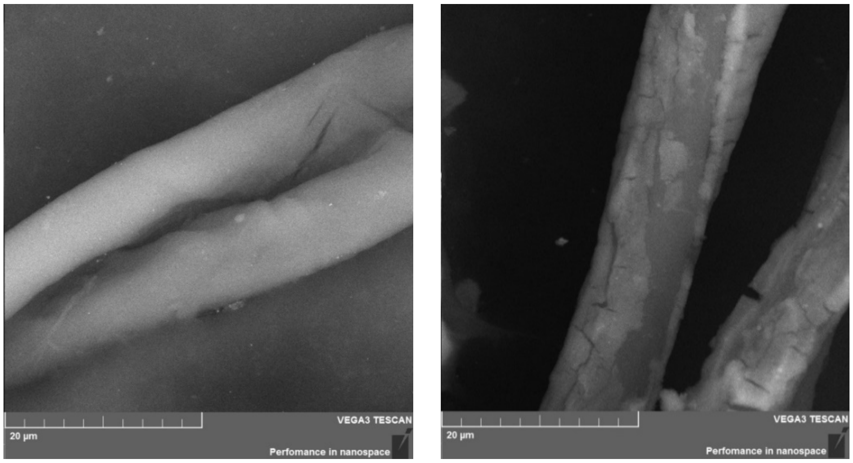

(a) (b)

Figure S6. SEM images of cotton (**a**) and cotton loaded with 0.25 mg/cm² TiO_2 (**b**) samples.

© 2020 by the authors. Submitted for possible open access publication under the terms and conditions of the Creative Commons Attribution (CC BY) license (http://creativecommons.org/licenses/by/4.0/).
